# Additional work of breathing from trigger errors in mechanically ventilated children

**DOI:** 10.1186/s12931-020-01561-3

**Published:** 2020-11-10

**Authors:** Robert G. T. Blokpoel, Alette A. Koopman, Jefta van Dijk, Martin C. J. Kneyber

**Affiliations:** 1 Department of Paediatrics, Division of Paediatric Intensive Care, Beatrix Children’s Hospital, University Medical Center Groningen, University of Groningen, Internal Postal Code CA 62, P.O. Box 30.001, 9700 RB Groningen, The Netherlands; 2 Critical Care, Anaesthesiology, Peri-Operative Medicine and Emergency Medicine (CAPE), University of Groningen, Groningen, The Netherlands

**Keywords:** Mechanical ventilation, Ineffective triggering, Work-of-breathing, Patient–ventilator asynchrony, Paediatric, Patient self-inflicted-lung injury

## Abstract

**Background:**

Patient–ventilator asynchrony is associated with increased morbidity and mortality. A direct causative relationship between Patient–ventilator asynchrony and adverse clinical outcome have yet to be demonstrated. It is hypothesized that during trigger errors excessive pleural pressure swings are generated, contributing to increased work-of-breathing and self-inflicted lung injury. The objective of this study was to determine the additional work-of-breathing and pleural pressure swings caused by trigger errors in mechanically ventilated children.

**Methods:**

Prospective observational study in a tertiary paediatric intensive care unit in an university hospital. Patients ventilated > 24 h and < 18 years old were studied. Patients underwent a 5-min recording of the ventilator flow–time, pressure–time and oesophageal pressure–time scalar. Pressure–time–product calculations were made as a proxy for work-of-breathing. Oesophageal pressure swings, as a surrogate for pleural pressure swings, during trigger errors were determined.

**Results:**

Nine-hundred-and-fifty-nine trigger errors in 28 patients were identified. The additional work-of-breathing caused by trigger errors showed great variability among patients. The more asynchronous breaths were present the higher the work-of-breathing of these breaths. A higher spontaneous breath rate led to a lower amount of trigger errors. Patient–ventilator asynchrony was not associated with prolonged duration of mechanical ventilation or paediatric intensive care stay.

**Conclusions:**

The additional work-of-breathing caused by trigger errors in ventilated children can take up to 30–40% of the total work-of-breathing. Trigger errors were less common in patients breathing spontaneously and those able to generate higher pressure–time–product and pressure swings.

**Trial registration:**

Not applicable.

## Background

Mechanical ventilation (MV) is one of the most common practiced interventions in the paediatric intensive care unit (PICU) [1]. In the absence of severe lung injury, there are several advantages associated with maintaining spontaneous breathing during MV including amongst others a lower need for sedation and a more even tidal volume (Vt) distribution towards the well-perfused lung-dependent zones thereby reducing shunting and lower lung inflammation [2–5].

When allowing for spontaneous breathing, it is imperative to achieve good interaction between patient demand and ventilator delivery. Patient–ventilator asynchrony (PVA) arises when the patient and ventilator are out-of-sync at any time point throughout the breathing cycle [6, 7]. It may lead to an increased use of sedatives and neuromuscular blocking agents, sleep disturbance, ventilator induced diaphragmatic dysfunction, and dynamic hyperinflation and volutrauma resulting from double triggering with subsequent breath stacking [8–13]. These detrimental effects may explain association between PVA and increased mortality and morbidity, albeit that a direct causative relationship has yet to be demonstrated [9, 14, 15].

**Fig. 1:**
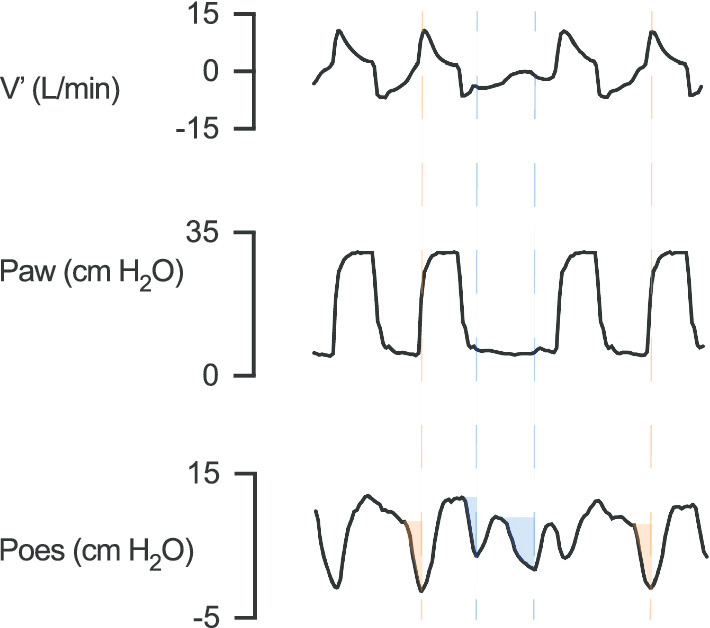
Example of ineffective and effective triggering in a mechanical ventilated child. Recording of airway pressure (Paw), oesophageal pressure (Poes) and ventilator flow (V’) versus time. Orange interrupted lines are showing effective triggering with in the orange shaded area an oesophageal pressure swing. Blue interrupted lines are showing ineffective triggering. Both ineffective errors are showing a different oesophageal pressure swing (blue area) with a concomitant different pressure–time–product (PTP) calculation

It has also been proposed that patients may experience increased work-of-breathing (WOB) related to PVA (WOB_PVA_), especially when there are trigger errors [16, 17]. This increased work comes from excessive pleural pressure swings (ΔP_pl_) generated during an inspiratory effort with subsequent additional lung stress, a phenomenon known as self-inflicted lung injury [18]. Two small studies in adults have shown that PVA can contribute up to 13–21% of the total WOB [19, 20]. Due to different respiratory mechanics these findings cannot be extrapolated to paediatrics. To date, it has not been studied if PVA in children is associated with increased WOB. Traditionally, total WOB is calculated using the Campbell diagram [21]. However, with ineffective triggering the flow generated by a patient is by definition insufficient to trigger the ventilator. Hence, the Campbell diagram cannot be constructed. The pressure–time product (PTP) may be used as WOB surrogate because it does not require any volume measurements but instead makes use of respiratory rate and duration of respiratory muscle contraction [21–23] (Fig. 1).

Previously, we reported that PVA is common in ventilated children, with ineffective triggering being the predominant type of PVA [24]. The objective of this exploratory study therefore was to calculate the added WOB_PVA_ caused by trigger errors in relation to total WOB by calculating the PTP, and to study the peak-to-through oesophageal pressure swing during ineffective and delayed triggering events.

## Methods

### Study population

This study was performed at the PICU of the Beatrix Children’s Hospital, University Medical Center Groningen. Patients ventilated > 24 h and < 18 years old able to trigger the ventilator were studied. Patients with neuromuscular disorders, premature birth with gestational age corrected for post-conceptional age less than 40 weeks, severe traumatic brain injury (i.e. Glasgow Coma Scale < 8), congenital or acquired damage to the phrenic nerve, congenital or acquired paralysis of the diaphragm, use of neuromuscular blockade, chronic lung disease (i.e. tracheostomy ventilation) and severe pulmonary hypertension were excluded. The Institutional Review Board waived the need for consent. Patients remained subjected to standard-of-care during the study (see Additional file 1).

### Data collection and variables

Patient baseline characteristics included age, gender, weight, admission diagnosis. Ventilator settings including mode, set positive end-expiratory pressure (PEEP) pressure above PEEP (PAP), mean airway pressure (Pmean), pressure support (PS), expiratory tidal volume (Vte ml/kg), mandatory breath rate, inspiratory time and fraction of inspired oxygen (FiO_2_) were recorded before start of the measurements. Clinical data included prior use of neuromuscular blockade (NMB), amount of analgesia-sedation in the 4 h preceding the recording, Comfort B score as measure of patient comfort, endotracheal tube (ETT) diameter and percentage of ETT leakage [25].

### Data acquisition and analysis

Ventilator settings were not changed during the study period unless the clinical condition of the patient dictated otherwise. Patients underwent a 5-min recording of the ventilator flow–time, pressure–time and oesophageal pressure–time scalar. Data were acquired through the Ventilator Open XML Protocol (VOXP) interface at a sampling rate of 100 Hz (Carefusion, Yorba Linda, CA, USA). All data was stored for offline analysis and subsequently processed using Polybench (Applied Biosignals GmbH, Weener, Germany).

For this study, we focused on ineffective and delayed triggering. First, we used three previously published studies to define the normal response time and trigger delay [26–28]. A normal response time was considered between 0 and 70 ms (ms) and a trigger delay was defined by a response time between 70 and 150 ms. Ineffective triggering (IT) was defined by the absence in ventilator pressurisation following a patient effort. Then, we identified IT and trigger delay in the recorded ventilator scalars. This is visualised by a simultaneous negative deflection in the pressure–time scalar, increase in the flow–time scalar and a negative deflection in the oesophageal–time scalar. We then calculated the trigger error index (TE-index) by the number of trigger error events (TEE) divided by the total number of breaths plus TEE times 100. Severe asynchrony was defined by TE-index > 10% and by TE-index > 75th percentile as proposed by others (i.e. TE-index > 22.5%) [14, 29].

PTP was calculated by integrating the area under the oesophageal pressure versus time scalar from the beginning until the end of inspiration [23, 30]. For each patient median PTP for effective and ineffective breaths were calculated. We determined for the entire 5-min recording of all effective (PTP_CUMULATIVE_BREATHS_) and ineffective breaths (PTP_CUMULATIVE_PVA_). PTP_TOTAL_ was defined as the sum of PTP_CUMULATIVE_BREATH_ and PTP_CUMULATIVE_PVA_. The oesophageal peak-to-trough _(_ΔP_oes_) was calculated by subtracting the end-inspiratory P_oes_ from the P_oes_ at the onset of inspiration.

We expected that patients with a lower number of ineffective triggering events would have lower PTP and ΔP_oes_. To compare the PTP between ineffective and effective breaths in each individual patient, the ratio of PTP_PVA_ over PTP_BREATH_ (PTP_PVA_/PTP_BREATH_) and ΔP_oes-ineffective_ over ΔP_oes-effective_ (ΔP_oes-ineffective_/ΔP_oes-effective_) was calculated.

### Statistical analysis

The Shapiro–Wilk test was used to test for normal distribution of the data. Normally distributed continuous data are presented as mean and standard deviation (SD). When the assumption of normality was not met, data are presented as median and 25–75 interquartile range (IQR). Categorical data are presented as percentage (%) of total. When comparisons between groups were made, continuous data were analysed using the Mann–Whitney *U* test. Spearman's rank correlation coefficient was used to measure dependence between two variables. All statistical analyses were performed using SPSS version 24 (IBM, Armonk, USA). *P* values below 0.05 were considered statistically significant.

## Results

In total 6194 breaths from 31 randomly selected patients (17 boys, 14 girls) were analysed. Median breaths during the 5-min recording was 180 [147; 249]. The median age was 3.0 [1.9; 18.5] months and median weight 5.6 [4.4; 9.8] kg. Median time patients were ventilated before data acquisition was 2.9 [1.9; 5.2] days. Median duration of MV was 5.9 [4.4; 9.5] days. NMB was used in 19 (61%) patients for a median duration of 31.8 [20.3; 51.2] hours. At the moment of data acquisition, NMB was stopped for a median duration of 25 [17.5; 48.9] hours. Twenty-three (74%) patients were admitted with primary respiratory failure, five (16%) after cardiac surgery, two (7%) for septic shock and one (3%) patient was admitted after trauma. Cuffed ETTs were used in 23 (74%) patients. Twenty-four (77%) patients were ventilated using pressure controlled (PC) / assist control (AC), 6 (19%) were supported with continuous positive airway pressure (CPAP) plus pressure support (PS) and one patient was on pressure-regulated volume control (PRVC/SIMV) + PS. During the recordings, median Vte was 6.9 [6.2; 7.6] mL/kg actual bodyweight, median end-tidal CO_2_ 6.42 [5.81; 7.18] kPa and median Comfort B score 12 [10; 12] (Table 1).

**Table 1:** Baseline demographics and ventilator settings

Variable	
N	31
Age (months)	3.0 [1.9; 18.5]
Weight (kg)	5.6 [4.4; 9.8]
Pulmonary diagnosis (n)	23
Surgical diagnosis (n)	5
Days on MV prior to study	2.9 [1.9; 5.2]
Duration of MV (days)	4.8 [3.6; 7.4]
Days on PICU	5.9 [4.4; 9.5]
NMB (h)	31.8 [20.3; 51.2]
NMB stopped prior to study (h)	25 [17.5; 48.9]
Cuffed endotracheal tube (%)	74
Comfort B score	12 [10; 12]
PAP (cm H_2_O)	16 [13; 20]
PEEP (cm H_2_O)	6 [5; 6]
Inspiration time (s)	0.6 [0.5; 0.68]
Set frequency (/min)	25 [20; 30]
Endtidal CO_2_ (kPa)	6.42 [5.81; 7.18]
Expiratory tidal volume (ml/kg)	6.9 [6.2; 7.6]

*MV* mechanical ventilation, *PICU* paediatric intensive care unit, *NMB* neuromuscular blockade, *PAP* pressure above PEEP, *PEEP* positive end expiratory pressure

Nine-hundred-and-fifty-nine trigger errors in 28 (90%) patients were identified, yielding a median TE-index of 9.7% [1.3; 22.5]. Patients had significantly lower TE-index when they were ventilated with a higher set inspiratory pressure (*r* = 0.537, *p* = 0.006), higher measured PIP (*r* = 0.644, *p* < 0.001) and higher Pmean (*r* = 0.435, *p* = 0.015). Patients had significantly lower TE-index if they had higher spontaneous breath rate (*r* = − 0.443, *p* = 0.13) and higher PTP_BREATH_ (*r* = − 0.365, *p* = 0.044).

The median PTP_CUMULATIVE_PVA_ was 4.7 cm H_2_O*s [0.5; 17.7]. The percentage of PTP_TOTAL_ caused by trigger errors was 11.5% [0.5; 34.3]. This percentage was significantly greater when patients were ventilated with higher set inspiratory pressures (*r* = 0.479, *p* = 0.015), PIP (*r* = 0.587, *p* = 0.001), Pmean (*r* = 0.383, *p* = 0.033) and higher mandatory breath rate (*r* = 0.667, *p* < 0.001), especially when there spontaneous breath rate was significantly lower (*r* = − 0.357, *p* = 0.049).

PTP_TOTAL_ significantly increased if patients were breathing more spontaneously (*r* = 0.489, *p* = 0.005) and mandatory breath rate was reduced (*r* = − 0.394, *p* = 0.029). Patients able to generate a higher PTP for a single effective breath (*r* = − 0.384, *p* = 0.033) and had higher levels of PTP_TOTAL_ (*r* = − 0.372, *p* = 0.039) spent less time on the ventilator.

Median ΔP_oes_ was 2.93 cm H_2_O [1.18; 5.56] when the triggering was effective and 1.94 cm H_2_O [0.69; 3.03] (*p* = 0.06) when there was a trigger error. This resulted in a median ΔP_oes-ineffective_ / ΔP_oes-effective_ of 0.79 [0.32; 1.03]. The median work patients generated during effective triggering (PTP_BREATH_) was 0.41 cm H_2_O*s [0.14; 1.01]. This was significantly higher compared with the work generated during ineffective triggering PTP_PVA_ (0.23 cm H_2_O*s [0.09; 0.53], *p* = 0.03). This resulted in a median PTP_PVA_/PTP_BREATH of_ 0.69 [0.17; 1.12]. We found that patients with a higher ΔP_oes-ineffective_/ΔP_oes-effective_ had a higher ITI (*r* = 0.512, *p* = 0.003) if they did not have spontaneous breaths outside the mandatory breath rate. ITI was significantly lower when patients had a total breath rate greater than the mandatory breath rate (ΔP_oes-ineffective_/ΔP_oes-effective_ *r* = − 0.577, *p* = 0.001). Similar observations were made for PTP_PVA_/PTP_BREATH_ (*r* = 0.541, [*p* = 0.002] and *r* = − 0.630 [*p* < 0.001] respectively).

### Subgroup analysis; severe asynchrony

**Fig. 2:**
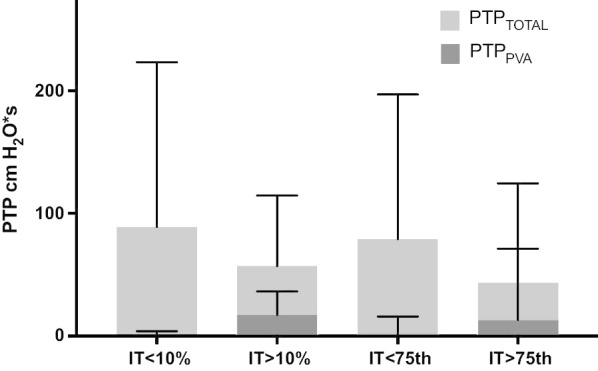
Distribution of percentage PTP_TOTAL_ caused by trigger errors in patients with severe asynchrony. Distribution of percentage PTP_TOTAL_ caused by trigger errors in patients with severe asynchrony. Severe asynchrony was defined as an ineffective triggering index (IT) > 10% and > 75th percentile

Analyzing the data set according to a paediatric and adult definition for severe asynchrony (i.e. TE-index > 75th percentile and > 10%) did not yield different results regarding patient discomfort, duration of MV or PICU stay [9, 30]. In addition, a subgroup analysis was made for patients who spend the highest amount of work-of-breathing on ineffective triggering (i.e. PTP_CUMULATIVE_PVA_ > 75th percentile, > 17.7 cm H_2_O*s) (Fig. 2). Subgroup analyses are shown in the online data supplement.

## Discussion

To our best knowledge this is the first study investigating the physiological effects of trigger errors in a heterogeneous group of ventilated children. Our main finding was that the additional work-of-breathing caused by trigger errors showed great variability among patients. Overall we found that the more asynchronous breaths were present the higher the work-of-breathing of these breaths. Our data also suggested that preserved respiratory muscle strength and higher spontaneous breath rate led to a lower amount of trigger errors. Yet, in our study PVA was not associated with prolonged duration of MV or PICU stay.

MV is initiated to reduce the respiratory muscle workload until the clinical condition of the patient has at least partially improved. However, there is limited data on acceptable levels of PTP in mechanically ventilated children. In healthy adults, PTP varies between 50 and 150 cm H_2_O*s/min [21]. Khemani et al*.* reported median PTP values of 41 cmH_2_O*s/min [9; 82] during + 10 cmH_2_O pressure support ventilation, 101 cmH_2_O*s/min [61; 165] on CPAP + 5 cmH_2_O and 135 cmH_2_O*s/min [84; 220] 5 min post-extubation without any positive pressure support in 409 children undergoing a spontaneous breathing trial (SBT) [31]. Others reported PTP 23 cmH_2_O*s/min [5; 89] before and 83 cmH_2_O*s/min [24; 110] during the SBT [30]. The PTP values observed in our study were lower than those previous reports. This might be explained by the fact that we also included patients early in the course of MV and not specifically during the weaning phase, thus our results may have been affected by the degree of respiratory muscle strength. We observed that that the additional energy expenditure from trigger errors was 11.5% [0.5; 34.3] and PTP_CUMULATIVE PVA_ of 4.7 cm H_2_O*s [0.5; 17.7]) during the 5-min recording. Taking the previously reported PTP values into consideration, the added work from trigger errors in our study may thus be interpreted as negligible and of little clinical importance [30, 31]. Nonetheless, we did find that the percentage of the additional work caused by trigger errors could reach up to 34–42% of energy expenditure albeit that the PTP values still remained low. Although this high percentage of wasted energy might be interpreted as unwanted, we could not demonstrate an association with adverse patient outcome.

There is also very little data on ΔP_oes_ in mechanically ventilated children. In our study ΔP_oes_ during trigger errors for the entire population and for the patients with severe PVA were below values Mortamet et al*.* and Rubin et al*.* described in paediatric population receiving MV [30, 32]. Because the ΔP_oes_ for trigger errors were below pleural pressure swings during conventional MV it may be supposed these pleural pressure swings did not contributed to patient self-inflicted lung injury. During the first 2 years of life there is a substantial reduction in chest wall compliance [33]. Hence, the question remains if paediatric patients can generate large pleural pressure swings, because of their compliant chest wall.

In our study, PTP_CUMULATIVE PVA_ increased with more asynchrony. In addition, with an increase in asynchrony we found that also PTP and ΔP_oes_ for an individual trigger error increased. These observations may have clinical implications. If trigger errors are merely detected using flow- and pressure—time tracings and not by measuring true patient effort using oesophageal pressure tracings, differentiation between “acceptable” and “harmful” trigger errors is not possible. This differentiation might be important, because the variability in PTP and ΔP_oes_ for an individual ineffective breath could partially explain that PVA has different effects on patient outcome. To illustrate, de Wit et al*.* and Blanch et al*.* described that PVA in the first 24 h and throughout MV was associated with prolonged ventilation time and mortality [14, 15]. In contrast, PVA during the weaning phase, using the same cut of values, was not associated with adverse clinical outcome [34]. It may be surmised that during the acute phase of disease causes patients generate more work and thus potentially injurious, larger pressure swings because respiratory system compliance (Crs) and respiratory muscle strength is reduced. Experimental work showed high pulmonary pressures swings generated by spontaneous breathing efforts worsened lung injury despite limiting plateau pressures [35]. When the clinical condition of the patient improves, the patient needs and is able to generate lower work to trigger the ventilator.

Some limitations of our study must be addressed. First, our data represents a single-center study, limiting generalizability. Second, in this study we found a lower TE-index than we previously did [24]. This is probably due to a difference in methodology to detect PVA. In our previous study we detected PVA using ventilator scalars without oesophageal pressure tracings, thereby probably overestimating the actual prevalence of PVA. Also, in the present study, patients were ventilated with a different ventilator brand with potentially differences in triggering response time [26]. Lastly, patients were randomly selected (as we had previously done), thereby potentially under- or overestimating TE. Third, we performed 5-min recordings. Because the occurrence of PVA is variable during the course of mechanical ventilation and even during the day we may have over- or underestimated the prevalence of trigger errors [15]. Last, our study mainly included patients younger than 1 year of age with relative higher respiratory rates, limiting extrapolation of our findings to older children and adults.

## Conclusion

The additional work-of-breathing caused by trigger errors in ventilated children can take up to 30–40% of the total work-of-breathing. Trigger errors were less common in patients breathing spontaneously and those able to generate higher PTP and pressure swings.

## Supplementary information

**Additional file 1.** Data supplement to; Additional work of breathing from trigger errors in mechanically ventilated children. Data containing the local ventilation guideline. Subgroup analysis of patients with an TE-index >75th percentile and PTPCUMULATIVE_PVA >75th percentile.

## Data Availability

The datasets analysed during the current study are available from the corresponding author on reasonable request.

## Abbreviations

AC: Assist control
CPAP: Continuous positive airway pressure
Crs: Respiratory system compliance
ETT: Endotracheal tube
IT: Ineffective triggering
ITE: Ineffective triggering events
IT-index: Ineffective triggering index
IQR: Interquartile range
MV: Mechanical ventilation
NMB: Neuromuscular blockade
PAP: Pressure above positive-end-expiratory pressure
PC: Pressure controlled
PEEP: Positive end-expiratory pressure
Pmean: Mean airway pressure
PICU: Paediatric intensive care unit
P_oes_: Oesophageal pressure
P_pl_: Pleural pressure
PRVC: Pressure-regulated volume control
PS: Pressure support
PTP: Pressure–time product
PTP_BREATH_: Pressure–time product of an individual effective breath
PTP_CUMULATIVE_BREATHS_: Pressure–time product of all effective breaths
PTP_CUMULATIVE_PVA_: Pressure–time product of all ineffective breaths
PTP_TOTAL_: Total pressure–time product
PTP_PVA_: Pressure–time product of an individual ineffective breath
PVA: Patient–ventilator asynchrony
SBT: Spontaneous breathing trial
SD: Standard deviation
TE-index: Trigger error index
TEE: Trigger error events
VOXP: Ventilator open xml protocol
Vt: Tidal volume
Vte: Expiratory tidal volume
WOB: Work-of-breathing
WOB_PVA_: Work-of-breathing related to patient–ventilator asynchrony
