# Supplementary material for: Additional work of breathing from trigger errors in mechanically ventilated children
Source: Respir Res. 2020 Nov 10;21:296. doi: 10.1186/s12931-020-01561-3 (PMC7653668; doi:10.1186/s12931-020-01561-3)
Supplement: Supplementary file 1 — Additional file 1. Data supplement to; Additional work of breathing from trigger errors in mechanically ventilated children. Data containing the local ventilation guideline. Subgroup analysis of patients with an TE-index >75th percentile and PTPCUMULATIVE_PVA >75th percentile. [file 12931_2020_1561_MOESM1_ESM.doc]

data supplement to

**Additional work of breathing from trigger errors in mechanically ventilated children.**

Robert G.T. Blokpoel, MD (1), Alette A. Koopman, MSc (1), Jefta van Dijk, MD (1), Johannes G.M. Burgerhof, MSc (2), Martin C.J. Kneyber, MD PhD FCCM (1, 3)

(1) Department of Paediatrics, Division of Paediatric Intensive Care, Beatrix Children's Hospital, University Medical Center Groningen, University of Groningen, Groningen, the Netherlands; (2) Department of Epidemiology, University Medical Center Groningen, University of Groningen, Groningen, the Netherlands; (3) Critical Care, Anesthesia, Peri-operative medicine & Emergency Medicine (CAPE), University of Groningen, Groningen, the Netherlands

*Ventilator protocol*

Patients were ventilated in a time-cycled, pressure-limited synchronized mode of ventilation with pressure support (PS). Children < 10 kg were ventilated using pressure controlled (PC) / assist control (AC), whereas children > 10 kg with lung injury were ventilated using PC /synchronized intermittent mandatory ventilation (PC/SIMV + PS) and those without lung injury using PC with preset tidal volume (Vt) with pressure support (PRVC/SIMV + PS). Patients were managed per a unit-based algorithm, targeting peak inspiratory pressure (PIP) < 28 – 32 cmH_2_O and expiratory Vt (Vte) 5 – 7 mL/kg actual bodyweight (since there was no obesity in this cohort). Vte was measured near the Y-piece of the endotracheal tube (ETT) in children < 10 kg (VarFlex^TM^, Vyaire, Mettawa, Ill, USA). Initial PEEP is 4-6 cm H_2_O in all patients, further titration is guided by the FiO_2_ required to maintain SpO_2_ 92 – 97% in the recovery phase and 88 – 92% during the acute phase. The mandatory breath rate was dictated by underlying respiratory mechanics and age to maintain pH > 7.20; the flow-time scalar is carefully observed in every patient when setting the inspiratory time and mandatory breath rate to prevent the development of intrinsic PEEP (PEEPi). The maximum I:E ratio is 1:1. The amount of pressure support in the PC/SIMV mode was PIP minus PEEP. Passive humidification by means of a heat-moisture exchanger was used in all patients on CMV (Gibeck, Teleflex Medical, Vianen, the Netherlands). Analgesia-sedation was maintained by a continuous infusion of midazolam, morphine, fentanyl or oral lorazepam. The COMFORT behavior scale was used to titrate the level of sedation .

*Subgroup analysis; TE-index > 10%*

Fourteen (45%) patients had a TE-index > 10%. Patients with a TE-index > 10% were younger of age (*p* = .044). Beside this baseline demographics and clinical demographics were comparable. Distribution of percentage PTP_TOTAL_ caused by trigger errors is shown in Figure 2. Patients with an elevated TE-index were ventilated with significantly higher PIP (*p* = .001) and Pmean (*p* = .019). The percentage of PTP_TOTAL_ caused by ineffective triggering and PTPCUMULATIVE_PVA was significantly higher in the group with an IT-index > 10% (*p* = .000) (*p* = .000). Patients with an IT-index < 10% were breathing more spontaneously (*p* = .002). A higher ratio ΔP_oes-ineffective_  / ΔP_oes-effective_ and ratio PTP_PVA_/PTP_BREATH_ between ineffective and effective breaths was seen in patients with an TEE-index >10% (*p* = .039) (*p* = .032). There were no differences in Comfort B score (*p* = .410), prior use of NMB (*p* = .238), duration of MV (*p* = .953) or PICU stay (*p* = .739), between the 2 groups.

*Subgroup analysis; TE-index >75^th^ percentile*

A subgroup analysis was made for patients with a TE-index > 75^th^ percentile (i.e.> 22.5%). Baseline and clinical demographics were comparable between the 2 groups. Patients with severe asynchrony were ventilated with significant higher inspiratory pressures (*p* = .041). The percentage of PTP_TOTAL_ caused by trigger errors and PTPCUMULATIVE_PVA were significantly higher in the group with a TE-index > 75^th^ percentile (*p* = .003) (*p* = .018). There were no differences in Comfort B score (*p* = .587), prior use of NMB (*p* = .271), duration of MV (*p* = .309) or PICU stay (*p* = .473), between the 2 groups.

*Subgroup analysis; PTPCUMULATIVE_PVA >75^th^ percentile*

A subgroup analysis was made for patients who spend the highest amount of work-of-breathing on ineffective triggering (i.e. PTPCUMULATIVE_PVA >75^th^ percentile, >17.7 cm H_2_O*s). Baseline demographics and clinical demographics were comparable between the 2 groups. PTP for a single trigger error was significantly higher in the PTPCUMULATIVE_PVA >75^th^ percentile group (*p* = .001). Patients with a PTPCUMULATIVE_PVA >75^th^ percentile were ventilated with higher levels of PIP and Pmean (*p* = .030) (*p* = .043). Median percentage of PTP_TOTAL_ caused by trigger errors was significantly higher in the PTPCUMULATIVE_PVA >75^th^ percentile group (*p* = .000). In addition the IT-index was higher in this group (*p* = .005). A higher ratio ΔP_oes-ineffective_  / ΔP_oes-effective_ and ratio PTP_PVA_/PTP_BREATH_ was seen in patients with a PTPCUMULATIVE_PVA >75^th^ percentile (*p* = .008) (*p* = .015). There were no differences between Comfort B score, duration of MV or PICU stay between the 2 groups.

**References**

1. Carnevale FA, Razack S, (2002) An item analysis of the COMFORT scale in a pediatric intensive care unit. Pediatr Crit Care Med 3: 177-180

2. Ambuel B, Hamlett KW, Marx CM, Blumer JL, (1992) Assessing distress in pediatric intensive care environments: the COMFORT scale. Journal of pediatric psychology 17: 95-109
